# Characteristics of Clinical and Electrophysiological Pattern in a Large Cohort of Chinese Patients With Charcot-Marie-Tooth 4C

**DOI:** 10.3389/fneur.2021.598168

**Published:** 2021-02-12

**Authors:** Xiaohui Duan, Yan Ma, Dongsheng Fan, Xiaoxuan Liu

**Affiliations:** ^1^Department of Neurology, China-Japan Friendship Hospital, Beijing, China; ^2^Department of Neurology, Peking University Third Hospital, Beijing, China; ^3^Key Laboratory for Neuroscience, Ministry of Education/National Health Commission, Peking University, Beijing, China

**Keywords:** Charcot-Marie-Tooth Disease, SH3TC2, phenotype, genotype, CMT4C

## Abstract

The “Src homology 3 (SH3) domain and tetratricopeptide repeats 2” (*SH3TC2*) gene is mutated in individuals with Charcot-Marie-Tooth disease (CMT) and considered relevant to a demyelinating or intermediate subtype of CMT disease, CMT4C. In this study, we screened a cohort of 465 unrelated Chinese CMT patients alongside 650 controls. We used Sanger, next-generation, or whole-exome sequencing to analyze *SH3TC2* and other CMT-related genes and identified 12 *SH3TC2* variants (eight novel) in seven families. Of the eight novel variants, seven were likely pathogenic (c.280–2 A > G, c.732–1 G > A, c.1177+6 T > C, c.3328–1 G > A, G299S, R548W, L1048P), and 1 had uncertain significance (S221P). The CMT4C frequency was calculated to be 4.24% in demyelinating or intermediate CMT patients without *PMP22* duplication. Additionally, we detected variant R954^*^ in the Chinese cohort in our study, indicating that this variant may be present among Asians, albeit with a relatively low frequency. The onset age varied among the eight patients, three of whom presented scoliosis. We summarized phenotypes in the Chinese CMT cohort and concluded that the absence of scoliosis, cranial nerve involvement, or late-onset symptoms does not necessarily preclude *SH3TC2* involvement in a given case.

## Synopsis

This study identified 7 novel, likely pathogenic variants of *SH3TC2* (c.280–2 A > G, c.732–1 G > A, c.1177+6 T > C, c.3328–1 G > A, G299S, R548W, L1048P), and the study results provide more insight into the diversity of CMT4C phenotypes.

## Highlights

- We identified 12 variants (eight novel) of the *SH3TC2* gene from 465 unrelated Chinese patients.
- The CMT4C frequency was calculated to be 4.24% in demyelinating or intermediate CMT patients without *PMP22* duplication.
- R954^*^ may be present among Chinese individuals, although its incidence may be low.
- CMT4C has diverse phenotypes, and the absence of scoliosis, cranial nerve involvement, or late-onset symptoms does not preclude *SH3TC2* involvement in a given case.

## Introduction

Charcot-Marie-Tooth disease (CMT) 4C is one of the most common forms of autosomal recessive (AR) demyelinating neuropathies caused by a mutation in the “Src homology 3 (SH3) domain and tetratricopeptide repeats 2” (*SH3TC2*) gene (1). The typical clinical features of CMT4C include early-onset distal motor and sensory neuropathy, scoliosis, and cranial nerve involvement. CMT4C progresses relatively slowly. Most patients can walk until they are 40–50 years old, indicating the prognoses of CMT4C are better than those of other AR CMT types, such as *GDAP1, PRX, MTMR2*, or *NDRG1*, which often cause an early loss of ambulation (2).

To date, more than 70 *SH3TC2* mutations have been identified in CMT4C patients (hihg.med.miami.edu/code/http/cmt/public_html/index.html#/). Although the majority of the mutations are nonsense mutations, missense, frameshift, and splicing mutations have also been reported. CMT4C is the most common form of AR CMT, with the prevalence varying from 1.38 to 26% among countries. The frequency of CMT4C among AR demyelinating CMT patients without *PMP22* duplication is >20% in the Czech Republic (3) and Italy (4) but is relatively low in Germany (5.2%) (5), Japan (1.76%) (6) and Korea (2.02%) (7). However, the frequency and clinical characteristics of CMT4C in mainland China remain unclear. A previous study screened for *SH3TC2* mutations in 84 Chinese probands with AR or sporadic CMT and identified only three novel heterozygous variants of undetermined significance (VUSs) (8). More detailed and reliable data based on a large cohort of Chinese patients are needed. Here, we describe the *SH3TC2* variants in a large cohort of Chinese patients. We aimed to investigate the phenotypic diversity in clinical or electrophysiological features, and the genotype-phenotype correlations among the patients, thereby providing new insight into the phenotypic spectrum of each genetic subgroup.

## Patients and Methods

### Patients

We enrolled 465 unrelated Chinese patients with CMT or related peripheral neuropathy between January 2007 and December 2019. The patients were classified based on their clinical phenotypes, mode of inheritance, and electrophysiological features. The family history, age of onset, clinical features, CMT neuropathy score (CMTNS), and electrophysiological features of the patients were also recorded in detail. The study was approved by the Ethics Committee of Peking University Third Hospital (IRB 00006761). Written informed consent was obtained from the patients or their parents for the publication of this report and any accompanying images.

### Mutation Analysis

Genomic DNA was extracted from the peripheral blood of the subjects using a DNA isolation kit (Bioteke, AU1802). Concentrations were determined with a Qubit fluorometer (Invitrogen, Q33216) and a Qubit dsDNA HS assay kit (Invitrogen, Q32851). Agarose gel (1%) electrophoresis was performed for quality control. The multiplex ligation-dependent probe amplification (MLPA) technique was performed in all patients with demyelinating and intermediate CMT, and 163 index patients with *PMP22* duplications and deletions were initially identified and excluded. From 2007 to 2013, *SH3TC2* mutations were screened by direct Sanger sequencing in 30 index patients suspected of having CMT. From 2014 to 2019, next-generation sequencing (NGS) gene panels covering 165 genes (Including SH3TC2) related to CMT and related diseases were performed in 202 index patients, and whole-exome sequencing (WES) was performed in 70 index patients. All suspected variants were validated by Sanger sequencing of *SH3TC2*.

#### Direct Sanger Sequencing

*SH3TC2* coding exons were amplified by polymerase chain reaction. The amplicons were analyzed using an ABI 3730XL DNA analyzer (Applied Biosystems, Waltham, MA, USA) in accordance with the manufacturer's protocol.

#### NGS

Sample dilution, flow-cell loading, and sequencing were performed in accordance with the Illumina specifications. DNA libraries were prepared with a KAPA library preparation kit (Kapa Biosystems, KR0453) in accordance with the manufacturer's instructions. To the capture probes and remove nonhybridized library components, pooled libraries were hybridized using the Agilent SureSelectXT2 target enrichment system. Molecular analysis was performed using a custom-designed targeted gene panel that covered 165 genes. The HiSeq2500 platform and 200-bp paired-end reads were used for sequencing.

#### WES

Agilent Human All Exon V6 kits were used for exome capture. The genes were sequenced on the HiSeq2500 platform with 200-bp paired-end reads.

### Pathogenicity Prediction

The pathogenicities of the CMT-related mutations were assessed using the standard method, which included phenotypic characterization; screening against dbSNP identifiers (http://www.ncbi.nlm.nih.gov/projects/SNP), 1,000 genomes (http://1000genomes.org/), and the ESP6500 and EVUS (http://evs.gs.washington.edu/EVS/) databases; a comparison with 650 Chinese controls; cosegregation with the phenotypes in the available familial cases; and *in silico* pathogenicity prediction by SIFT (http://sift.jcvi.org/www/SIFT_enst_submit.html), PolyPhen (http://genetics.bwh.harvard.edu/pph2/index), and Mutation Taster (http://www.mutationtaster.org/). The splice-site mutations were predicted using the software HSF 3.0 (https://www.genomnis.com/access-hsf). Variant classification was based on the American College of Medical Genetics (ACMG) standards (2015) (9), and the pathogenic or likely pathogenic variants were identified accordingly.

## Results

### Clinical Data

The genotypic and phenotypic analyses in the cohort of 465 CMT index patients yielded diagnoses of 276 CMT1, 137 CMT2 and 52 intermediate CMT. After 163 index patients with *PMP22* duplication or deletion and 137 patients with CMT2 were excluded, the remaining 165 patients were classified as demyelinating or intermediate CMT patients without *PMP22* duplication. *SH3TC2* mutations were detected in 7 of these 165 index patients (4.24%); 2 of these patients were included in the 42 AR families (4.76%), and five were included in the 123 (4%) isolated patients.

Ten patients from seven families were included in genetic testing, but the clinical features were only obtained from eight patients with *SH3TC2* mutations (Table 1). All of the patients had symptoms of foot drop, pes cavus and walking difficulties. The mean age of onset was 21.1 years and ranged from 4 to 43 years. Only one patient experienced her first symptom in the first decade of life. Four were between 10 and 20 years old. The ages of onset for the other three patients were 35, 40, and 43 years. The initial symptoms included foot drop, walking difficulties, muscle weakness in the distal lower limbs, scoliosis, and pes cavus. The mean disease duration was 10.4 years, with a duration range from 2 to 24 years, and the progression rate was mild or modest, as evidenced by the CMTNS (with a range of 8–18). Upper and lower extremity areflexias were detected in all of the patients. Three of eihgt patients had hearing loss (Family 2 II1, family 6 III3 and family 7 II1). Other types of cranial involvement, such as nystagmus or facial paresis, were not observed. Prominent scoliosis was observed in three of the eight patients. In patient II1 from family 4, the disease began with scoliosis, whereas scoliosis appeared later, after motor and sensory distal neuropathies developed, in patient II3 from family 2 and II1 from family 3 Proprioceptive ataxia was observed in two patients (Family 2 II1 and family 3 II1). Six of eight patients had decreased vibratory sensation, including one patient had totally absent vibratory sensation leading to severe proprioceptive ataxia. Sensory loss was more severe in vibration than in pain sensation. Only one patient (III3 from family 6) had motor predominant neuropathy with relatively normal sensory test clinically and electrophysiologically. This patient is 35-year-old women, who suffered from walking difficulty due to foot drop for 2 years. She also reported hearing loss. Her brother and twin sister had similar symptom, but refused to come to the hospital to do physical and electrophysiological examinations.

**Table 1:** Clinical features of the patients in this study.

**Families/patients**	**Onset age**	**Mode of inheritance**	**Disease duration**	**Sclerosis**	**Hearing loss**	**Muscle strength**		**Muscle atrophy**		**Sensory pinprick**	**Vibration**	**CMTNS**	**Clinical features**
						**APB/FDI/ADM**	**Dorsiflexion/plantar flexion**	**UL P/D**	**LL P/D**				
Fam1II1	10	Isolated	5	–	–	5–/5/5–	2/4	–/–	+/++	–	↓up to knee	13	Clumsy gait, Pes Cavus CK 520
Fam2II1	43	AR	8	–	–	5–/5–/5–	2/3	–/–	–/+	–	↓up to knee	12	Foot drop, clumsy gait
Fam2II3	40		4	+	+	5/5/5	2/1	–/–	+/+++	–	N	16	Clumsy gait, Ataxia
Fam3II1	10	Isolated	17	+	–	4/4/3	1/3	–/+	+/+++	↓up to knee	Absent	14	Ataxia, Pes Cavus
Fam4II1	4	Isolated	6	+	–	4/4/5-	3/3	–/–	+/++	–	↓below ankle	12	Clumsy gait
Fam5II1	12	Isolated	24	–	–	3/3/3–	2/2	+/+	+/++	–	↓below ankle	16	Distal motor and sensory neuropathy
Fam6III3	35	AR	2	–	+	5/5/5	3/4	–/–	+/++	–	N	8	Foot drop, walking difficulty, motor predominant
Fam7II1	15	Isolated	17	–	+	5–/5/5	1/2	–/–	+/++	–	↓below ankle	18	Foot drop, clumsy gait

*APB, abductor pollicis brevis; FDI, first dorsal interosseous; ADM, abductor digiti minimi; UL, upper limb; LL, lower limb; Muscle atrophy severities: + mild atrophy; ++ moderate atrophy; +++ severe atrophy;=. Sensory examination: N, normal; ↑, increased; ↓, decreased*.

*+, positive; –, negative; P/D, proximal/distal*.

### Electrophysiological Results

Electrophysiological studies revealed a sensorimotor demyelinating neuropathy in all the individuals. The motor and sensory nerve conduction velocity (NCV) was low and within a range indicating demyelinating and intermediate CMT. The NCV results tended to be more severe in the lower limbs than in the upper limbs, while the sensory nerves tended to show more severe effects than did the motor nerves. The mean motor conduction velocity (MCV) was 30.8 m/s in the median and ulnar nerves (range, 21.6–41 m/s). Three patients (Fam1 II1, Fam2 II3, and Fam 6 III3)showed mildly delayed MCVs (range, 31–41 m/s). The mean compound muscle action potential (CMAP) was 3.0 mV (range, 0.7–6.8 mv) in the median and ulnar nerves. The mean MCV was 24.9 m/s in the peroneal and tibial nerves (range, 16.3–33.2 m/s). The CMAP of the peroneal nerve was absent in five of the eight patients. The mean CMAP was 0.7 mV (range, 0.3–1.6 mV) in the peroneal nerve of the remaining patients. The sensory conduction velocities (SCVs) of the median, ulnar, and sural nerves were not elicited in four patients. The mean SCV was 35.7 m/s in the median and ulnar nerves (range, 21–50 m/s) and 30 m/s in the sural nerve (range, 10–41.1 m/s). The mean sensory nerve action potential (SNAP) of the sural nerve was 4.9 μV (range, 3–6.4 μV). Only one index patient (III3 from family 6) had motor-predominant symptoms with only minor sensory involvement; the sural SNAP was 6 μV, while the peroneal CMAP was absent (Table 2).

**Table 2:** Neurophysiologic data of the patients in this study.

**Families/patients**	**Motor nerve conductions**												**Sensory nerve conductions**								
	**Median**			**Ulnar**			**Tibial**			**Peroneal**			**Median**			**Ulnar**			**Sural**		
	**DL ms**	**Amp.mv**	**CV, m/s**	**DL, ms**	**Amp.mv**	**CV, m/s**	**DL, ms**	**Amp.mv**	**CV, m/s**	**DL, ms**	**Amp.mv**	**CV, m/s**		**SNAP, uv**	**CV, m/s**		**SNAP, uv**	**CV, m/s**		**SNAP, uv**	**CV, m/s**
Fam1II1	6.2	4.1	31.6	5.0	3.8	31.5	9.7	2.3	25.7	9.4	1.6	25.2	Abs	Abs	Abs	Abs	Abs	Abs	Abs	Abs	Abs
Fam2II1	8.7	0.7	22.7	5.3	4.1	34.5	Abs	Abs	Abs	Abs	Abs	Abs	Abs	Abs	Abs	Abs	Abs	Abs	Abs	Abs	Abs
Fam2II3	7.0	5.0	31	4.5	7.8	41	Abs	Abs	Abs	Abs	Abs	Abs	3.3	7	37	2.7	10	36	4.1	4	32
Fam3II1	8.5	2.4	21.6	7.6	3.0	24.9	10.9	1.4	24.5	12.3	0.3	17.4	Abs	Abs	Abs	Abs	Abs	Abs	Abs	Abs	Abs
Fam4II1	22.2	0.8	17	25.6	1.1	15	27.3	0.5	10	16.3	0.5	11	6.5	2	21	5.4	7	22	12.1	3	10
Fam5II1	10.7	1.0	27.4	7.4	2.4	29.3	7.2	3	29.5	Abs	Abs	Abs	Abs	Abs	Abs	Abs	Abs	Abs	Abs	Abs	Abs
Fam6III3	5.1	3.0	40.7	5.9	5	40.9	6.8	2.3	26.1	Abs	Abs	Abs	2.6	5.4	39	2.1	8	50	2.5	6	41.1
Fam7II1	6.7	6.8	28.1	4.4	3.3	40.4	7.3	0.8	33.2	Abs	Abs	Abs	3.4	9.4	36.4	2.5	8.8	44.4	2.1	6.4	36.8

*Abs, absent*.

*DL, distal latency; Amp, amplitude; CV, conduction velocity; SNAP, sensory nerve action potential*.

### SH3TC2 Mutation Analysis

Four pathogenic (E553^*^, E657K, R904^*^, and R954^*^) and 7 likely pathogenic (c.280–2 A > G, c.732–1 G > A, c.1177+6 T > C, c.3328–1 G > A, G299S, R548W, L1048P) *SH3TC2* variants and 1 *SH3TC2* VUSs (S221P) were detected among the seven families. Eight of these variants were novel mutations, and most of them were nonsense or splice-site mutations, which have been suspected of causing the premature termination of translation or the production of nonfunctioning proteins. To determine the pathogenicity of the *SH3TC2* variants, a bioinformatics tool was utilized. The variants were classified according to the standards and guidelines of the ACMG (9). The results of the *in silico* analysis and predicted pathogenicity of these variants are illustrated in Table 3.

**Table 3:** Molecular analysis results and predicted pathogenicities of the variants in this study.

**Family**	**Exon**	**Nucleotide change**	**AA change**	**Effect**	**Database**			**Pathogenicity**			**GERP**	**Evidence**	**ACMG**	**Chromosomal location**	**HGMD/ClinVar**
					**Exac**	**1,000G**	**650 controls**	**SIFT**	**Polyphen-2**	**Mutation taster**				**(chr5)**	
Fam1II1	11	c.1969 G>A	p.E657K		0.00001647	–	0	Damaging	Probably damaging	Damaging	6.16	PS1 + PM2 + PP3 + PS3	Pathogenic	148407326	HGMD (1)
	7 Intron6	c.732-1 G>A	Splicing		–	–	0			Damaging	5.68	PVS1 + PM2	Likely Pathogenic	148420241	
Fam2II1	11	c.1642 A>T	p.R548W	TPR1	–	–	0	Damaging	Probably damaging	Damaging	3.7	PM1 + PM2 + PP1 + PP3 + PP4	Likely Pathogenic	148407653	
Fam 3II1	11	c.2710 C>T	p.R904*		0.00005765	–	0	/	/	Damaging	6.02	PVS1 + PM2	Pathogenic	148406585	HGMD (2)
	13	c.3143 T>C	p.L1048P		0.000008236	0.000199681	0	Damaging	Probably damaging	Damaging	5.67	PM1 + PM2 + PP1 + PP3	Likely Pathogenic	148392208	
Fam 4II1	11	c.2860 C>T	p.R954*		0.0008813	–	0	/	/	Damaging	4.13	PVS1	Pathogenic	148406435	HGMD (1)
	15 Intron14	c.3328-1 G>A	Splicing	TPR5	–	–	0	/	/	Damaging	6.03	PVS1 + PM2	Likely Pathogenic	148388565	
Fam 5II1	10 Intron11	c.1177+6 T>C			–	–	0	–	–	–	–	PM2 + PM3 + PM4 + PP3	Likely Pathogenic	148408234	
	11	c.1657 G>T	p.E553*	TPR1	–	–	0			Damaging	6.04	PVS1 + PM2	Pathogenic	148407638	HGMD (10)
Fam 6III3	8	c.895 G>A	p.G299S	SH3 2	–	–	0	Damaging	Probably damaging	Damaging	4.88	PM2 + PP3	Likely Pathogenic	148417964	
	11	c.1657 G>T	p.E553*	TPR1	–	–	0			Damaging	6.04	PVS1 + PM2	Pathogenic	148407638	HGMD (10)
Fam 7II1	6	c.661 T>C	p.S221P	SH3 1	0.000008236	–	0	Tolerated	Probably damaging	Damaging	5.6	PM2	VUS	148421049	
	4 Intron3	c.280-2 A>G	Splicing		–	–	0	?	?	Damaging	5.92	PVS1 + PM2	Likely Pathogenic	148424203	

*Transcript version: NM_024577.3*.

**Figure 1:**
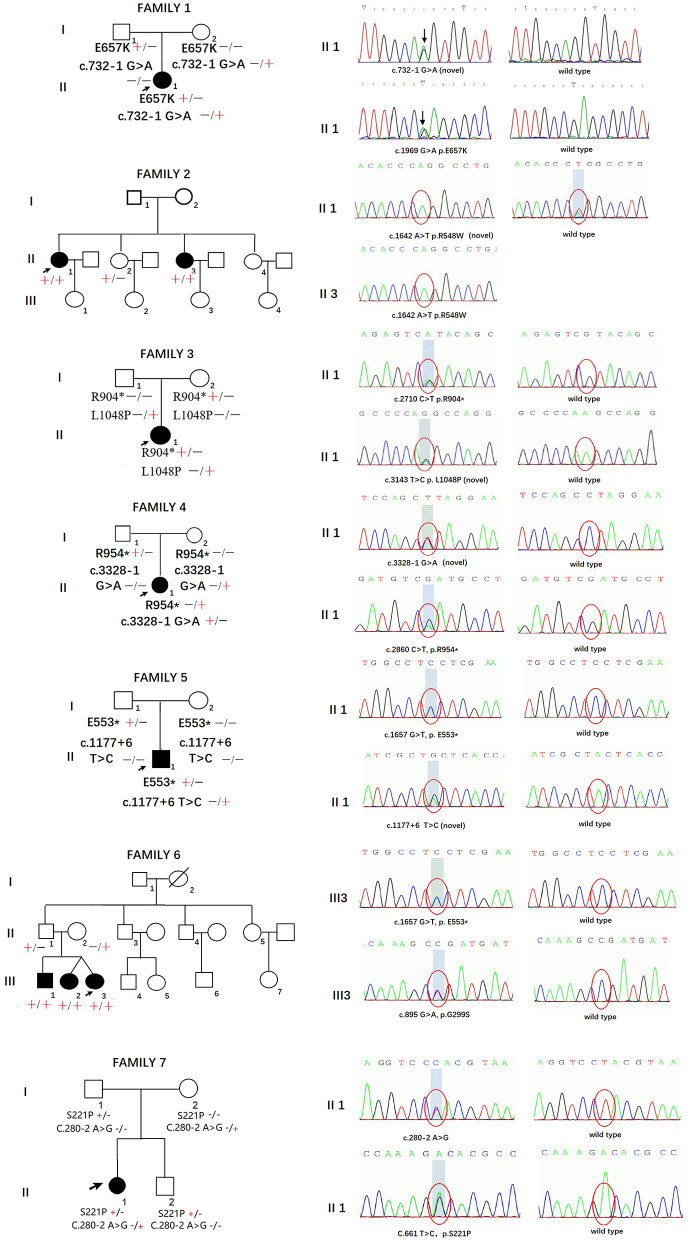
Pedigrees and genotypes of seven families in our study with novel *SH3TC2* variants.

**Figure 2:**
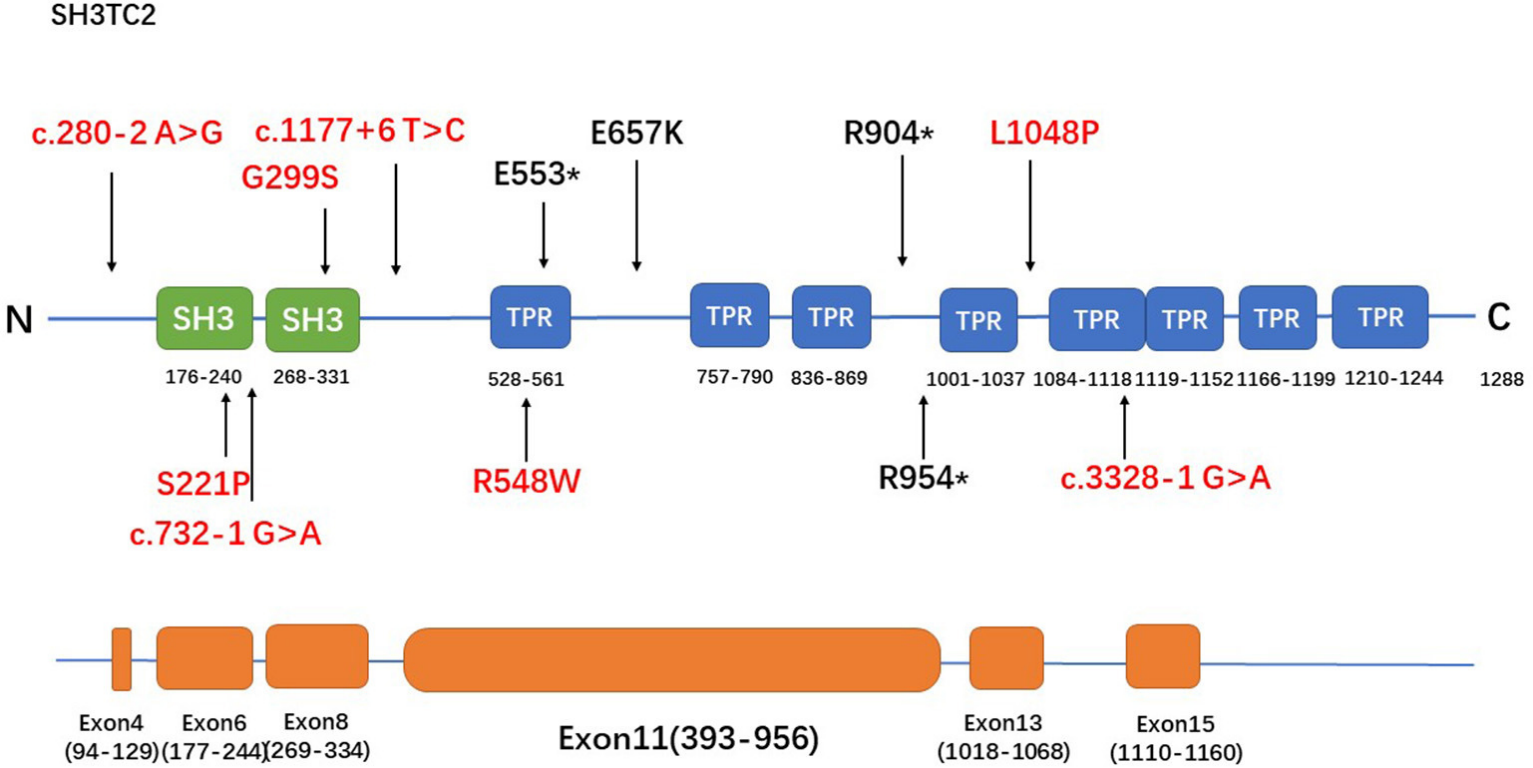
The spectrum of SH3TC2 mutations identified in this study. The upper panel is the SH3TC2 protein structure (Uniprot: Q8TF17) and the bottom panel shows the SH3TC2 gene sequence, showing exons associated with identified mutations. Five of the 12 mutations lie in exon 11, and the remaining mutations are scattered along the whole gene. Novel mutations were labeled in red.

The pedigrees and genotypes of the families who carried the novel *SH3TC2* variants are illustrated in Figure 1. The proband from family 1 carried E657K and c.732–1 G > A as compound heterozygous mutations, and they were inherited from her healthy mother and father, respectively. The mutation c.732–1 G > A is a novel splicing mutation. The E657K mutation has previously been reported to be a pathogenic mutation (1). The proband from family 2 carried the novel homozygous missense mutation R548W. This mutation was also found in her sister, who had a similar demyelinating neuropathy and ataxia; however, this mutation was not found in her other two healthy sisters, and thus segregated with other family members. The heterozygous compound novel mutations L1048P and R904^*^ in *SH3TC2* were detected in the proband from family 3. L1048P has never been reported before, whereas R904^*^ is described to be pathogenic in the Human Gene Mutation Database (HGMD) database (2). Both parents, each of whom carried a variant, were found to have normal clinical and electrophysiological results. The proband from family 4 carried the previously reported R954^*^ (1) and the novel splicing mutation c.3328–1 G > A as compound heterozygous mutations. The proband from family 5 carried the novel splicing mutations c.1177+6 T > C and E553^*^ as compound heterozygous mutations. The proband from family 6 was found to be heterozygous for the *SH3TC2* nonsense mutation E553^*^ (10) and novel missense mutation G299S. The proband from family 7 was found to be heterozygous for the *SH3TC2* novel missense mutation S221P and splicing mutation c.280–2 A > G. The mutation S221P was defined as a VUS according to the ACMG classification, whereas c.280–2 A > G was classified as a likely pathogenic mutation. The locations and distributions of *SH3TC2* variants in our study are presented in Figure 2.

## Discussion

CMT4C was first recognized in 1996, and *SH3TC2* was first identified as the causative gene of CMT4C by Senderek et al. (1) in 2003. To date, over 70 *SH3TC2* mutations have been confirmed. These mutations include nonsense, splice-site, and frameshift mutations, either in homozygous or compound heterozygous states, and they lead to the premature termination of protein synthesis or the production of nonfunctional proteins. Some missense mutations, such as E657K, R1109P, and R648W, have also been observed (1, 2, 11). In our study, 3 previously reported nonsense mutations (E553^*^, R904^*^, and R954^*^) (1, 10, 12), 4 novel splicing mutations (c.280–2 A > G, c.732–1 G > A, c.3328–1 G > A, c.1177+6 T > C), and 5 missense mutations, including 1 that was previously reported (E657K) (1), 3 that are likely pathogenic (R548W, L1048P, G299S) and 1 that is VUS (S221P), were identified. Only one homozygous mutation (R548W) was found in this series. The remaining six index patients had varied compound heterozygous mutations comprising the mutations indicated above.

CMT4C has been reported to be the most common form of AR CMT. In this study, the CMT4C frequency was calculated to be 4.24% (7/165) in demyelinating or intermediate CMT patients without *PMP22* duplication, 2.13% (7/328) in demyelinating and intermediate CMT patients with *PMP22* duplication, and 1.5% (7/465) in the whole CMT cohort in our study. The frequency in demyelinating or intermediate CMT patients without *PMP22* duplication is slightly higher in this Chinese cohort than in patients in other Asian countries, such as Japan (1.76%) (6) and Korea (2.02%) (7). For American and European counties, the frequency of CMT4C in the Chinese CMT cohort in our study (1.5%) was similar to or slightly higher than those in Germany (1.7%) (5), the United States (0.8%) (13), and the United Kingdom (0.6%) (14). In a Southern Italy study, the prevalence of SH3TC2 in overall CMT population was 3.2%, but it raised at 11.6% in sporadic demyelinating CMT cohort (SH3TC2 represents the second prevalent gene in this selected population) (15).

The R954^*^ mutation, which is located in exon 11, has been reported to be particularly prevalent in central Europe, the Mediterranean basin, and the USA (1, 3, 5, 11, 15–18). This mutation has been identified in more than half or nearly all of the patients in English, French, Greek, Norwegian and Czech cohorts (3, 11, 16–18), whereas none of the patients in Japanese and Korean cohorts had reported the mutation (6, 7). The R954^*^ mutation was also found in the population in our study with a relatively low frequency (1/12). The R904^*^ mutation, another relatively frequently occurring mutation in *SH3TC2* (2, 6), was also identified in the population in our study. A study involving a Greek cohort (11) reported that most of the cases are caused by the p.R1109^*^ mutation. This founder mutation found in European Gypsies (2, 19) has also been found in pure Japanese populations (6) but was not detected in the population in our study. Five of the 12 mutations detected in our study lie in exon 11, and the remaining mutations are scattered along the whole gene (Figure 2). This tendency might be attributed to the fact that exon 11 is the largest exon of the gene and accounts for approximately half of the coding sequence.

Clinically, CMT4C has been described to be a slowly progressive disorder with an early onset, and spine scoliosis and cranial involvement are frequently observed as well. The age of onset in the cohort in our study varied and was not as young as those previously reported (1, 12, 15, 16). Three of the eight patients experienced symptoms in adulthood. There are several studies suggesting that CMT4C has a late onset (6, 11, 20). A recent study from Greece reported one patient who did not have symptoms until the age of 55 years (11). In a large French-Canadian kindred, the average age of diagnosis was reported to be 35.8 years (21). The six affected siblings in the kindred showed variable rates of progression of the symptoms, indicating a broad spectrum of phenotypes and complicated genotype-phenotype correlations in patients with CMT4C. The progression rate was mild or modest in the cohort in our study, as evidenced by the CMTNS (range, 8–18). None of the patients required a wheelchair at the last follow-up.

Regarding scoliosis, spinal deformities are often described within the first decade of life and considered hallmark signs of CMT4C (2). However, in our study, only three of the eight patients presented with scoliosis. Scoliosis was not detected in the other five patients, even after their CT scans were examined, suggesting that the absence of scoliosis cannot rule out a diagnosis of CMT4C. Azzedine et al. (12) noted that nearly all Algerian patients who present with scoliosis are homozygous for the R954^*^ or R904^*^ mutation. The two patients with scoliosis in our study carried R954^*^ and R904^*^ as compound heterozygous mutations in each allele. The low prevalence of R954^*^ or R904^*^ may partly explain the relatively low frequency of scoliosis in the cohort in our study. However, not all CMT4C patients with spinal deformities carried the R954^*^ or R904^*^ mutation. Patient II3 **from family 2** with the homozygous R548W mutation in our study showed scoliosis after the onset of peripheral neuropathy. Moreover, one patient from the homozygous R954^*^ Dutch family did not present with scoliosis (12), suggesting that additional genetic or environmental factors may contribute to the expression of this clinical feature. Although the pathophysiology of this type of pronounced scoliosis is not fully understood, spinal deformities can be caused by motor deficits in the paravertebral muscles, and the *SH3TC2* protein might be involved in spine development (17).

We also observed that three of the eight patients presented with hearing loss. This symptom is not specific and thus cannot distinguish CMT4C from the other forms of demyelinating CMT, such as CMT1A, 1B, and X1, caused by mutations in *PMP22, P0*, or *GJB1*. Although cranial nerve involvement is commonly observed in CMT4C patients, we surprisingly did not detect any other cranial nerve involvement. The study by Colomer et al. (2) reported cranial nerve involvement in 9 of the 15 cases. The authors described hearing loss, slow pupillary reflexes, and tongue fasciculation to be the main features of cranial nerve involvement. Another study by Kontogeorgiou et al. (11) reported that 4 of 13 index patients presented with cranial nerve involvement. The most commonly affected nerve is the acoustic nerve, followed by the trigeminal, facial, vagus and hypoglossal nerves. Therefore, cranial nerve involvement is a supportive symptom of CMT4C, but the absence of cranial nerve symptoms should not exclude *SH3TC2* from consideration as a potential determinant in a given CMT4C case.

Due to the above findings, considerable variability in phenotype, disease severity, and disease duration were observed in our study, despite the presence of identical genetic mutations. Such as E553^*^ mutation has been identified both in family 5 and family 7, but the patient from family 5 had more severe deep sensation involvement, while the patient from family 7 had prominent cranial involvement, such as hearing loss. Electrophysiological studies also showed broad variability. Unlike the homogenous decline of MCV in patients with CMT1A, nearly half patients presented with intermediate decline of MCV in median and ulnar nerve, which might mimic the NCVs results of CMTX patients. Although sensory never are more often affected in CMT4C patients, motor predominant with minor sensory involvement can be also observed. Another study from a Korea cohort also supported this finding, which reported the sural SNAP was 7–7.1 μV with mildly reduced MCV (36.9–38 m/s), while the peroneal CMAP was severely declined in 2 affected patients in CMT4C (7). Yuan JH et al. even reported 2 of 8 patients presented axonal neuropathy in their NCS results (6). These findings confirmed the NCS potentiality of widening the phenotypical spectrum of CMT disease. Recent mutations in EGR2 gene (classically related to demyelinating CMT) was associated to an axonal phenotype (22). Taken together, NCS studies can give us insight to better understanding the underlying pathophysiological mechanism of CMT4C.

The *SH3TC2* protein contains 8 tetratricopeptide repeat (TPR) domains and 2 SH3 domains. Proteins with TPR domains are responsible for many cellular functions, such as RNA synthesis, axonal transport, and chaperone functions, through protein-protein interactions. SH3 domains are highly conserved. They play important roles in intracellular communication and signal transduction. *SH3TC2* is exclusively expressed in Schwann cells and has been shown to target recycling endosomes by associating with the small GTPase Rab11 (23). The function of *SH3TC2* and the effects of *SH3TC2* mutations should be further investigated. We predicted that the majority of the mutations in *SH3TC2* cause the production of truncated *SH3TC2* proteins or the *SH3TC2* protein to be missing due to nonsense-mediated mRNA decay. Accordingly, our observations suggest that loss of function in *SH3TC2* may be the underlying cause of AR mutations. Otherwise, the genetic modifier of *SH3TC2*, such as common SNPs in *SH3TC2* or transcription regulator (SOX10/CREB) might be responsible for the broad variability in CMT4C (24).

In summary, the CMT4C frequency was calculated to be 4.24% in demyelinating or intermediate CMT patients without *PMP22* duplication. We identified 4 novel splice sites and 3 novel missense mutations in *SH3TC2* to be likely pathogenic. R954^*^ and R904^*^ were also detected in the Chinese population, but the frequency was relatively low. A broad spectrum of phenotypes was observed in the Chinese CMT cohort in our study. The absence of scoliosis, cranial nerve involvement, or late-onset symptoms does not preclude *SH3TC2* involvement in CMT cases.

## Data Availability Statement

All the data and material in this article are publicly available and can be found at http://www.mono-mybg.com/jzjycmt.

## Ethics Statement

The studies involving human participants were reviewed and approved by the Institutional Ethics Committee of Peking University Third Hospital (PUTH) approved this study (IRB 00006761). Written informed consent to participate in this study was provided by the participants' legal guardian/next of kin.

## Author Contributions

XD conceived and designed the study. DF provided valuable clinical materials. XL performed the genetic testing and reviewed and edited the manuscript. XD and XL wrote the paper. All authors contributed to the article and approved the submitted version.

## Conflict of Interest

The authors declare that the research was conducted in the absence of any commercial or financial relationships that could be construed as a potential conflict of interest.
